# Supplementary material for: Current Therapeutic Strategies for Metastatic Triple-Negative Breast Cancer: From Pharmacists’ Perspective
Source: J Clin Med. 2022 Oct 12;11(20):6021. doi: 10.3390/jcm11206021 (PMC9604829; doi:10.3390/jcm11206021)
Supplement: Supplementary file 1 [file jcm-11-06021-s001.zip › jcm-1856351-Supplementary.pdf]

Journal of Clinical Medicine

Review

Current Therapeutic Strategies for Metastatic Triple-Negative Breast Cancer: From Pharmacists' Perspective

Shuanghe Li [1,2], Chongyang Bao [1,2], Lingli Huang [1,*] and Ji-Fu Wei [1,3,*]

[1] Department of Pharmacy, Jiangsu Cancer Hospital (Jiangsu Institute of Cancer Research, Nanjing Medical University Affiliated Cancer Hospital), Nanjing 210009, China
[2] Department of Clinical Pharmacy, School of Basic Medicine and Clinical Pharmacy, China Pharmaceutical University, Nanjing 211198, China
[3] Department of Clinical Pharmacy, School of Pharmacy, Nanjing Medical University, Nanjing 211103, China
* Correspondence: lingli_huang@njmu.edu.cn (L.H.); weijifu@njmu.edu.cn (J.-F.W.)

Search strategies for Web of science, Pubmed, Embase, and Cochrane databases

Pubmed

1. Search (((((Triple negative breast cancer[Title/Abstract]) OR (TNBC[Title/Abstract])) OR (HER2-negative breast cancer[Title/Abstract])) OR (metastatic triple negative breast cancer[Title/Abstract])) OR (HER2-negative metastatic breast cancer[Title/Abstract])) OR (MBC[Title/Abstract])
2. (((clinical trial[Publication Type]) OR(Randomized controlled trial [Publication Type])) OR (randomized [Title/Abstract])) OR placebo

After we determined agents to be discussed, the search strategies based on drugs related were as follows:
1. Search (((((Triple negative breast cancer[Title/Abstract]) OR (TNBC[Title/Abstract])) OR (HER2-negative breast cancer[Title/Abstract])) OR (metastatic triple negative breast cancer[Title/Abstract])) OR (HER2-negative metastatic breast cancer[Title/Abstract])) OR (MBC[Title/Abstract])) OR (breast cancer[Title/Abstract])) OR (BC[Title/Abstract])
2. Search ((utidelone[Title/Abstract]) OR (UTD1[Title/Abstract])
3. Search (eribulin[Title/Abstract]) OR (E7389-LF[Title/Abstract]))OR(eribulin mesylate[Title/Abstract])
4. Search (((sacituzumab govitecan[Title/Abstract]) OR (sacituzumab govitecan-hziy[Title/Abstract])) OR (trodelvy[Title/Abstract])) OR (SG[Title/Abstract])
5. Search (olaparib[Title/Abstract]) OR (lynparza[Title/Abstract])
6. Search ((((talazoparib[Title/Abstract]) OR (talazoparib tosylate[Title/Abstract])) OR (BMN-673[Title/Abstract])) OR (talzenna[Title/Abstract]))
7. Search ((pembrolizumab[Title/Abstract]) OR (keytruda[Title/Abstract]))
8. Search (atezolizumab[Title/Abstract]) OR (Tecentriq[Title/Abstract])
9. Search ((((tratuzumab deruxtecan[Title/Abstract]) OR (ENHERTU[Title/Abstract])) OR (DS-8201[Title/Abstract])) OR (T-DXd[Title/Abstract])) OR (fam-trastuzumab deruxtecan-nxki[Title/Abstract])
10. (((clinical trial[Publication Type]) OR(randomized controlled trial [Publication Type])) OR (randomized [Title/Abstract])) OR placebo

Web of science

1. TS= ("Triple negative breast cancer" OR "TNBC" OR "HER2-negative breast cancer" OR "metastatic triple negative breast cancer" OR "HER2-negative metastatic breast cancer" OR "MBC")
2. TS = ("random controlled trial" OR "random" OR "placebo")

After we determined agents to be discussed, the search strategies based on drugs related were as follows:
1. TS= ("Triple negative breast cancer" OR "TNBC" OR "HER2-negative breast cancer" OR "metastatic triple negative breast cancer" OR "HER2-negative metastatic breast cancer" OR "MBC" OR"breast cancer" OR "BC")

2. TS= (“utidelone” OR “UTD1”)
3. TS= (“eribulin” OR “E7389-LF” OR “eribulin mesylate”)
4. TS= (“sacituzumab govitecan” OR “sacituzumab govitecan-hziy” OR “trodelvy” OR “SG”)
5. TS= (“olaparib” OR “lynparza”)
6. TS= (“talazoparib” OR “talazoparib tosylate” OR “BMN-673” OR “talzenna”)
7. TS= (“pembrolizumab” OR “keytruda”)
8. TS= (“atezolizumab” OR “tecentriq”)
9. TS= (“tratuzumab deruxtecan” OR “ENHERTU” OR “DS-8201” OR “T-DXd” OR “fam-trastuzumab deruxtecan-nxki”)
10. TS = (“random controlled trial” OR “random” OR “placebo”)

Embase

1. “Triple negative breast cancer”:ab,ti OR “TNBC”:ab,ti OR “HER2-negative breast cancer”:ab,ti OR "MBC":ab,ti OR "metastatic triple negative breast cancer":ab,ti
2. “random”:ab,ti OR “placebo”:ab,ti OR “double-blind”:ab,ti

After we determined agents to be discussed, the search strategies based on drugs related were as follows:
1. “Triple negative breast cancer”:ab,ti OR “TNBC”:ab,ti OR “HER2-negative breast cancer”:ab,ti OR "MBC":ab,ti OR "metastatic triple negative breast cancer":ab,ti OR “breast cancer”:ab,ti OR “BC”ab,ti
2. “utidelone”:ab,ti OR “UTD1”:ab,ti
3. “eribulin”:ab,ti OR “E7389-LF”:ab,ti OR “eribulin mesylate”:ab,ti
4. “sacituzumab govitecan”:ab,ti OR “sacituzumab govitecan-hziy”:ab,ti OR “trodelvy”:ab,ti OR “SG”:ab,ti
5. “olaparib”:ab,ti OR “lynparza”:ab,ti
6. “talazoparib”:ab,ti OR “talazoparib tosylate”:ab,ti OR “BMN-673”:ab,ti OR “talzenna”:ab,ti
7. “pembrolizumab”:ab,ti OR “keytruda”:ab,ti
8. “atezolizumab”:ab,ti OR “tecentriq”:ab,ti
9. “tratuzumab deruxtecan”:ab,ti OR “ENHERTU”:ab,ti OR “DS-8201”:ab,ti OR “T-DXd”:ab,ti OR “fam-trastuzumab deruxtecan-nxki”:ab,ti
10.“random”:ab,ti OR “placebo”:ab,ti

Cochrane Library

1. "Triple negative breast cancer":ti,ab,kw OR "TNBC":ti,ab,kw OR "HER2-negative breast cancer":ti,ab,kw OR "MBC":ti,ab,kw OR "metastatic triple negative breast cancer":ti,ab,kw

After we determined agents to be discussed, the search strategies based on drugs related were as follows:
1. "Triple negative breast cancer":ti,ab,kw OR "TNBC":ti,ab,kw OR "HER2-negative breast cancer":ti,ab,kw OR "MBC":ti,ab,kw OR "metastatic triple negative breast cancer":ti,ab,kw OR "breast cancer":ti,ab,kw OR "BC":ti,ab,kw
2. "utidelone":ti,ab,kw OR "UTD1":ti,ab,kw
3. "eribulin":ti,ab,kw OR "E7389-LF":ti,ab,kw OR "eribulin mesylate":ti,ab,kw
4. "sacituzumab govitecan":ti,ab,kw OR "sacituzumab govitecan-hziy":ti,ab,kw OR "Trodelvy":ti,ab,kw OR "SG":ti,ab,kw
5. "olaparib":ti,ab,kw OR "lynparza":ti,ab,kw
2. "talazoparib":ti,ab,kw OR "talazoparib tosylate":ti,ab,kw OR "BMN-673":ti,ab,kw OR "Talzenna":ti,ab,kw
3. "pembrolizumab":ti,ab,kw OR "keytruda":ti,ab,kw OR
4. "atezolizumab":ti,ab,kw OR "tecentriq":ti,ab,kw
5. "tratuzumab deruxtecan":ti,ab,kw OR "ENHERTU":ti,ab,kw OR "DS-8201":ti,ab,kw OR "T-DXd":ti,ab,kw OR "fam-trastuzumab deruxtecan-nxki":ti,ab,kw
